# Improved Soil Temperature Modeling Using Spatially Explicit Solar Energy Drivers

**DOI:** 10.3390/w10101398

**Published:** 2018

**Authors:** Jonathan J. Halama, Bradley L. Barnhart, Robert E. Kennedy, Robert B. McKane, James J. Graham, Paul P. Pettus, Allen F. Brookes, Kevin S. Djang, Ronald S. Waschmann

**Affiliations:** 1 Western Ecology Division, National Health and Environmental Effects Research Laboratory, U.S. Environmental Protection Agency, Corvallis, OR 97330, USA;; 2 College of Earth and Atmospheric Sciences, Oregon State University, Corvallis, OR 97331, USA;; 3 Environmental Science & Management, Humboldt State University, Arcata, CA 95521, USA;; 4 Inoventures (LLC), Western Ecology Division, National Health and Environmental Effects Research Laboratory, c/o U.S. Environmental Protection Agency, Corvallis, OR 97330, USA;

**Keywords:** soil temperature, solar energy, watershed model, landscape scale, VELMA

## Abstract

Modeling the spatial and temporal dynamics of soil temperature is deterministically complex due to the wide variability of several influential environmental variables, including soil column composition, soil moisture, air temperature, and solar energy. Landscape incident solar radiation is a significant environmental driver that affects both air temperature and ground-level soil energy loading; therefore, inclusion of solar energy is important for generating accurate representations of soil temperature. We used the U.S. Environmental Protection Agency’s Oregon Crest-to-Coast (O’CCMoN) Environmental Monitoring Transect dataset to develop and test the inclusion of ground-level solar energy driver data within an existing soil temperature model currently utilized within an ecohydrology model called Visualizing Ecosystem Land Management Assessments (VELMA). The O’CCMoN site data elucidate how localized ground-level solar energy between open and forested landscapes greatly influence the resulting soil temperature. We demonstrate how the inclusion of local ground-level solar energy significantly improves the ability to deterministically model soil temperature at two depths. These results suggest that landscape and watershed-scale models should incorporate spatially distributed solar energy to improve spatial and temporal simulations of soil temperature.

## 1. Introduction

Soil temperature affects several key ecosystem properties. Through surface runoff and subsurface groundwater transport, soil temperatures can lead to increased stream temperatures, which in turn impact salmonid and other fish habitats [1]. Soil temperatures mediate rates of biogeochemical transformations in soils, strongly influencing local to global-scale patterns in the cycling, retention and loss of carbon and nutrients from ecosystems [2]. Seasonal soil temperature trends can shift photosynthetic recovery timing and therefore impact overall net primary production (NPP) [3]. Such soil temperature effects are subject to modification by physical landscape factors, such as object shading, slope aspect and thermal isolation from a detritus layer or snow pack [4].

Mechanistic watershed models such as Visualizing Ecosystem Land Management Assessments (VELMA) (2.0, U.S. Environmental Protection Agency—Western Ecology Division, Corvallis, OR, USA) [5], Soil andWater Assessment Tool (SWAT) (2009, Texas A&M, College Station, TX, USA) [6], Regional Hydro-Ecologic Simulation System (RHESSys) (2004, University of California, Santa Barbara, CA, USA) [7], and Hydrologic Simulation Program—Fortran (HSPF) (11, U.S. Environmental Protection Agency—National Exposure Research Laboratory, Athens, GA, USA) [8] use a mechanistic (as opposed to statistical) approach to model hydrodynamics throughout a watershed using sub-daily or daily time steps. Models such as these utilize equations to simulate hydrologic dynamics and soil moisture by tracking the rate of water transfer based on soil porosity, soil depth and the available precipitation.

Watershed models simulating soil temperature at multiple depths rely on several observed and simulated environmental variables including air temperature, precipitation, soil moisture, soil depth, and physical soil properties. For national and regional-scale modeling purposes, climate data can often be obtained from various governmental agencies such as the National Oceanic and Atmospheric Administration (NOAA), Natural Resources Conservation Service (NRCS) and others that maintain large-scale networks of climate monitoring stations such SNOTEL and SCAN [9]. At more local scales, climate data collection tends to focus on site-specific requirements, such as municipal airports, Long Term Ecological Research Stations and university research forests [10–13]. This can result in data limitations for spatially explicit models [14]. Several groups process the site data to produce spatial datasets at various spatial scales (e.g., Parameter-elevation Regressions on Independent Slopes Model (PRISM) and Daily Surface Weather Data (Daymet)) [15,16]. Soil column properties can be acquired through field work or obtained by utilizing soil datasets (e.g., State Soil Geographic (STATSGO) and Soil Survey Geographic (SURGO)) [17].

While climate and soil properties influence soil temperatures, solar energy is the most significant environmental variable influencing soil temperature. There are existing spatial models that account for solar energy inputs at a local or stream reach scale: SHADE2 (1.0, University of Georgia, Athens, GA, USA) [18], HeatSource (8.0, Oregon Department of Environmental Quality, Portland, OR, USA) [19], and iLand (1.0, Seidl and Rammer, Vienna, Austria) [20], utilize small-area representations of solar energy. However, these model’s spatial heterogeneity of shade is utilized for quantifying shade along stream reaches or within forest plots; not ground-level irradiance within models representing complete watersheds or landscapes.

Previous methods of incorporating solar energy within model representations of complete watersheds employ one or more proxy variables (e.g., canopy coverage or air temperature), or they simply utilize an average daily global irradiance value. Current environmental mechanistic models (e.g., VELMA, SWAT, RHESSys, HSPF) [5–8] use a global solar irradiance subroutine to calculate the total solar energy (W/m^2^) for the entire watershed or for sub-catchments. Due to their simpler ground-level solar energy representations, although these models capture the seasonal pattern of irradiance, they lack a spatially heterogenous representation of topographic and landscape object shading that affects ground-level solar energy levels. Solar energy estimate methods themselves include uncertainty due to environmental variables like cloud fraction and albedo [21]. Additional variables (i.e., aerosol optical properties, cloud asymmetry, water vapor distribution) may have seasonal and regional influence on the accuracy of solar energy estimates [22].

There remains a gap in soil temperature modeling where current approaches utilize global solar energy models that are not capturing local energy interactions. Finer-scale spatially distributed estimates of ground-level shade or solar energy could provide improved soil temperature estimates. This paper addresses this gap by incorporating local solar energy data within the soil temperature subroutine of an ecohydrological watershed model to determine its effect on simulated soil temperature predictions at multiple depths.

To demonstrate the utility of linking spatially explicit, ground-level solar energy data with an environmental model, we focus on improving VELMA’s soil temperature subroutine by incorporating spatially heterogeneous solar energy as an input driver. First, we discuss a commonly used global solar energy model and soil temperature model that are found in many deterministic watershed models, including VELMA. Next, we present VELMA’s original soil temperature subroutine that incorporates spatially explicit inputs of soil moisture and air temperature but does not employ any form of solar energy presentation. Following the original model, we then present VELMA’s modified soil temperature subroutine that incorporates spatially explicit inputs of ground-level solar energy, along with the inclusion of soil moisture and air temperature. We compare the predictive skill of the original and new forms of VELMA’s soil temperature subroutines using multiple United States Environmental Protection Agency (EPA) Oregon Crest-to-Coast Environmental Monitoring Transect (O’CCMoN) sites. This transect consists of several paired sites of forested and open landscapes [23]. We present results that demonstrate the benefit of including spatially explicit representations of solar energy within watershed-scale models that simulate soil temperature.

## 2. Materials and Methods

Watershed models typically include solar energy directly or through a proxy variable to facilitate energy requirements needed within subroutine routines. Plant growth models may require a daily input of solar energy reaching the canopy to drive photosynthesis [24]. Stream temperature models predict shifts in water temperature through variables representing landscape shading, water temperatures, and air temperatures, all of which are solar energy proxies. Snowmelt models may need a daily input of solar energy or air temperature to drive snow melt [25]. While all these subroutines rely on solar energy at the earth’s surface, mechanistic models generally lack the ability to capture the spatiotemporal dynamics of solar energy reaching the ground post shadowing.

The soil temperature subroutine from VELMA [5] was chosen for testing. VELMA is a spatially distributed watershed model that simulates hydrologic and biogeochemistry processes within a gridded framework under mechanistic cell interactions. Using a gridded framework, VELMA describes each grid cell as having a ground-level surface and four sub-surface voxels representing the landscapes soil strata. Each subsurface voxel is characterized by soil porosity and soil depth. Water transfers at a daily time step through VELMA’s voxel framework. Based on water transmission, nutrients and thermal energy migrate through the simulated soil substrate under mechanistic rules.

### 2.1. Previous Solar and Generalized Soil Modeling Methods

Watershed models often employ a clear-sky solar energy model when direct solar energy units are required. A common approach, and the method used by VELMA, is to calculate the clear-sky solar energy that reaches the earth’s surface as in Equation (1), where *R* is solar irradiance (W/m^2^), *ecc* is the eccentricity correction factor, *w* is the Earth’s constant angular velocity, *T* is the time frequency, *dec* is the solar declination, and *γ* is latitude [26]:

(1) R=(24/π)×4.921×ecc×[wT×sin(dec)×sin(γ)+cos(dec)×sin(wT)×cos(γ)]

The VELMA model uses Equation (1) to describe the amount of solar energy reaching the troposphere under clear sky conditions. This approach does not account for the topographic or object shading that locally reduces ground-level solar energy.

Soil temperature modeling within many watershed models, whether utilizing a gridded representation of the landscape or aggregating to sub-catchment scales, typically uses some version of the Carslaw and Jaeger equation to quantify seasonal variation in soil temperature [27]:

(2) $$T_{soil}\left(z,d_{n}\right)=T_{AA}+A_{surf}\times {\text{e}}^{-z/dd}\times \sin\left(\omega \times d_{n}-z/dd\right)$$

where *T_soil_*(*z*, *d_n_*) is the soil temperature (°C) at depth *z* (mm) for day of the year *d_n_*, *T_AA_* is the average annual soil temperature, *A_surf_* is the amplitude of the surface fluctuations, *dd* is the damping depth (mm), and ω is the angular frequency of the damping oscillations by day (*d_n_*). At *z* = 0, the soil temperature reduces to the following:

(3) $$T_{soil}\left(0,d_{n}\right)=T_{AA}+A_{surf}\times \sin\left(\omega \times d_{n}\right)$$

which is the average soil temperature perturbed by surface temperature fluctuations and reflects seasonal solar patterns. Conversely, at infinite depth, the soil temperature becomes equal to the annual average soil temperature. This formulation provides a relatively simple method for calculating soil temperatures at multiple depths throughout a watershed. However, the model requires specification of soil heat capacity as well as thermal conductivity to correctly specify the amplitude coefficient and the damping depth.

### 2.2. Soil Temperature Variations Due to Landscape Coverage

**Figure 1.**
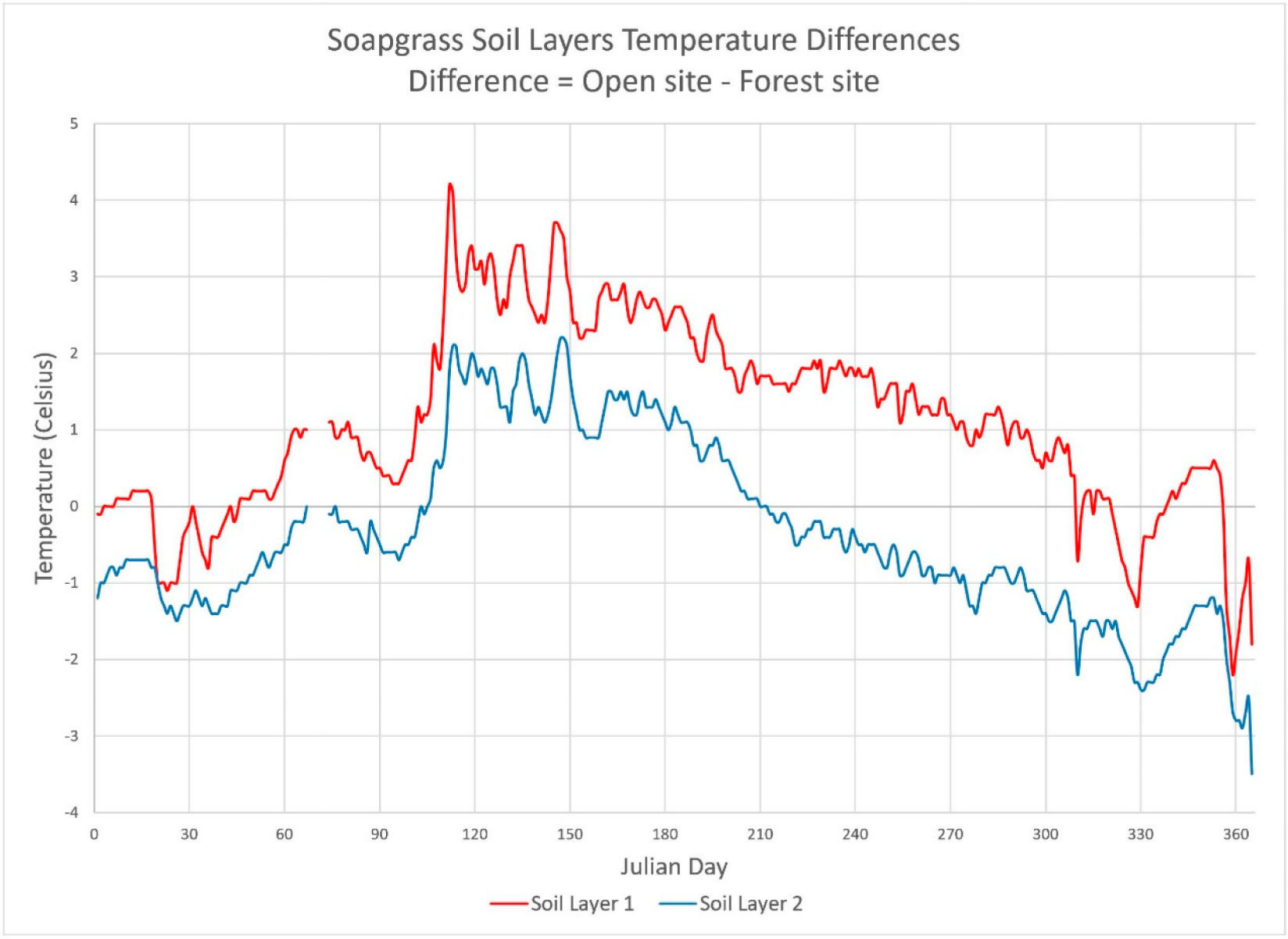
Daily differences in soil temperature (°C) between open (clear-cut) and forest sites at the O’CCMon Soapgrass site. Temperature differences were calculated as the Open Site temperature minus the forest site temperature at each Julian day during the year 2005. Thus, positive values denote days where the open site soil temperature was warmer than the forest site soil temperature. Open minus forest soil temperature differences were calculated for each of the two soil depths, 15 cm (red line) and 30 cm (blue line) below the soil surface. The data gap between Julian days 67 and 74 was due to sensor errors.

Temperature profiles of soils can dramatically vary between a forested versus open environment, even if the sites are located proximally near one another. Two sites can be exposed to very similar climate conditions, though due to forest canopy shading, the forested site will have reduced air temperature and a reduction in solar energy loading upon the soil surface. Figure 1 shows observed 2005 daily soil temperature differences between open (clear-cut harvested) site data minus forested site data for the O’CCMoN Soapgrass field site in Oregon (see Section 2.5.1 for field site locations).

Figure 1 highlights the soil temperature differences between open and forested sites. A positive temperature means the open site temperature was warmer than the forest site; conversely, negative temperature means the open site temperature was colder than the forest site. Two main observations should be made here: (1) layer 1 is always warmer than layer 2, and (2) for both soil layers the open site is always warmer in summer than the forest site, but is comparatively colder in winter and especially so in layer 2. The open site is significantly warmer from Julian day 45 through 310 (14 February through 6 November), with a peak difference of 4.2 °C on Julian day 111 (21 April).

**Figure 2.**
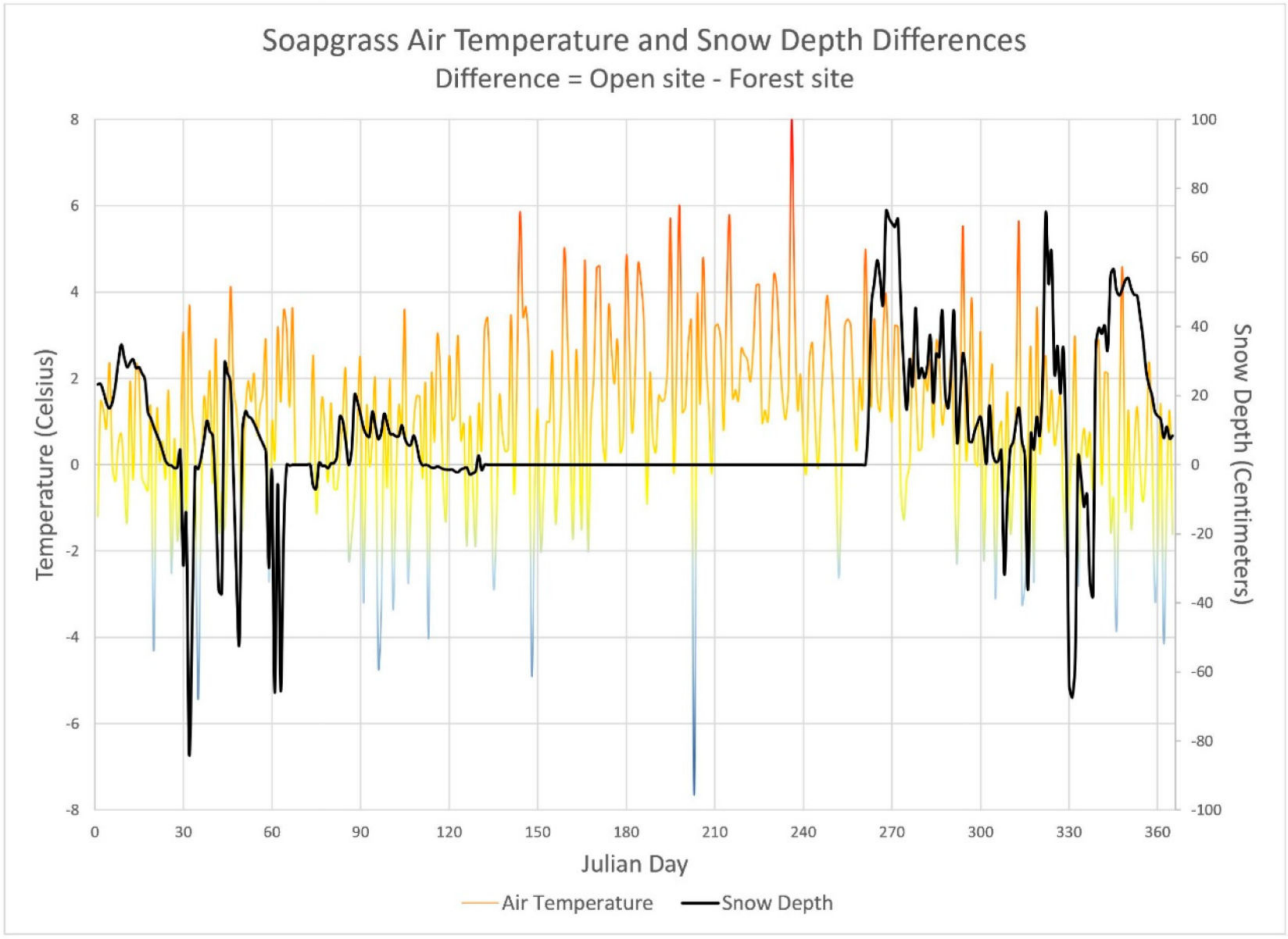
Daily air temperature (°C) and snow depth (cm) for both the forest and open sites at the Soapgrass station. A positive air temperature difference means the open site was warmer than the forest site. A positive depth difference means the open site had more snow than the forest site.

Seasonal differences in the warming and cooling of soils in the open and forest sites (Figure 1) certainly reflect changes in air temperature along with some complicating effects associated with inter-site variations in snow pack and associated insulative properties (Figure 2). Other factors undoubtedly also come into play, such as the effects of seasonal changes in soil moisture (dry summers, wet winters) on soil thermal transmissivity.

Nevertheless, observed increases in summer air temperatures for the open site tended to be 2.12 °C warmer than the forest site (Figure 2). The lowest thermal difference was −7.65 °C while the highest thermal difference was 8.0 °C. During the same period, soil layer 1 open site averaged 1.71 °C warmer than the forest site with a minimum of 1.0 °C and a maximum of 2.6 °C. However, soil layer 2 open site averaged −0.16 °C cooler than the forest site with a minimum of −1.0 °C and maximum of 1.3 °C. That is, while air temperature differences are driven by differences in solar radiation, in the open site there is clearly an additional direct effect of solar radiation on heat transfer to the ground surface and consequent warming of the soil column. This observation underscores the importance of quantifying the direct effect of solar radiation on soil temperature, in combination with effects of soil moisture and other factors mentioned above.

### 2.3. Original VELMA Air Soil Temperature (AST) Subroutine

**Figure 3.**
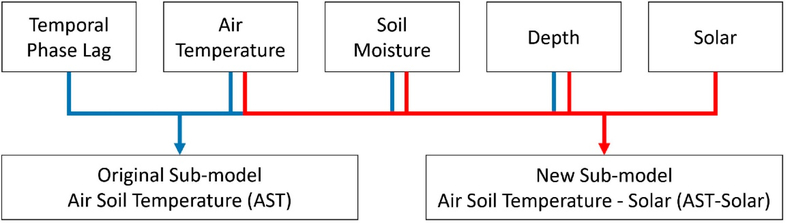
Combined AST and AST-Solar subroutines’ schematic depicts the input data driving both models and displays the crucial data input difference between AST and AST-Solar with Temporal Phase Lag influencing only AST and Solar Energy influencing only AST-Solar.

For calculating spatially distributed soil temperature, VELMA accounts for soil moisture damping and the oscillatory effects of solar energy through a modified version of the Carslaw and Jaeger equation. This approach accounts for the seasonal solar energy variability through a time phase lag modification of observed air temperature combined with a temperature modification based on a soil depth attenuation. VELMA’s subroutine, along with the equation previously presented by Carslaw and Jaeger (1959) (Equations (2) and (3)), does not account for spatial heterogeneity of solar energy reaching the ground due to topographic or object shading; instead, VELMA’s input variables account for the shift in soil temperature due to daily air temperature, soil moisture and soil depth (Figure 3).

For each layer, the AST subroutine calculates the soil temperature based on the thermal attenuation of daily air temperature. The input variables for the AST and AST-Solar models are mostly the same but might be utilized differently within each subroutine’s equation setup, with the exceptions of the Air_LAG_ and Reducer_SOLAR_(Table 1).

The degree of attenuation is adjusted daily by the depth and soil moisture of each soil layer. G_TEMP_ is the resulting soil temperature due to: Air_AVETEMP_ being the daily average air temperature (Table 1), Air_LAG_ (Equation (5) from a prior Air_AVETEMP_ (Table 1) based on seasonal oscillation, Soil_DAMPING_ (Equation (7) influencing the soil moisture based on seasonal oscillation, Depth_ATTENUATION_ (Equation (9) based on soil depth and soil damping (Equation (7), Phase_LAG_ (Equation (6) influenced by the Air_AVETEMP_ (Table 1) based on seasonal oscillation driven by LS_DEPTH_ (depth to surface) (Table 1), Soil_DAMPING_ (Equation (7), LTD (Equation (8) being the soil temperature accumulation at depth, and Soil_BELOW_ (Table 1) influencing soil temperature from the lower soil layer:

(4) $${\text{G}}_{\text{TEMP}}={\text{Air}}_{\text{AVETEMP}}+({\text{Air}}_{\text{LAG}}-{\text{Air}}_{\text{AVETEMP}}-{\text{Soil}}_{\text{DAMPING}})\times {\text{Depth}}_{\text{ATTENUATION}}$$

(5) $${\text{Air}}_{\text{LAG}}=\text{Past}\;\text{Air}\;\text{Temperature}\;\text{at}\;\text{Julian}\;{\text{Day}}^{\prime }\text{s}\;{\text{Phase}}_{\text{LAG}}$$

(6) $${\text{Phase}}_{\text{LAG}}=\left({\text{LS}}_{\text{DEPTH}}/{\text{Soil}}_{\text{DAMPING}}\right)\times (365/2\pi )$$

(7) $${\text{Soil}}_{\text{DAMPING}}\text{=}\sqrt{\frac{\text{LTD}\times \text{365}}{\pi }}$$

(8) $$\text{LTD}={\text{LTD}}_{\text{ACCUMULATION}}/{\text{Soil}}_{\text{BELOW}}$$

(9) $${\text{Depth}}_{\text{ATTENUATION}}={\text{e}}^{\wedge }\left(-{\text{LS}}_{\text{DEPTH}}/{\text{Soil}}_{\text{DAMPING}}\right)$$

Each day, the air temperature is given as an input for each cell within VELMA’s watershed framework. The Soil_DAMPING_ (Equation (7) and LS_DEPTH_ (Table 1) variables are used to dampen the variations of soil temperature at larger depths. Any soil temperature shifts due to solar energy are incorporated via a proxy of two oscillatory equations driven by past air temperature (Equation (5) and soil moisture damping (Equation (7). VELMA AST subroutine’s performances for open and forested landscapes are tested in the model testing section below.

### 2.4. New VELMA Air Soil Temperature-Solar (AST-Solar) Subroutine

The previously described soil temperature model does not utilize spatially explicit solar energy data, so we improved the model by adding the capacity to utilize spatially distributed ground-level solar energy to the current VELMA AST subroutine. This new model is called Air Soil Temperature-Solar (AST-Solar). The inclusion of solar energy within VELMA’s original AST model was mainly accomplished through the addition of the new parameter Reducer_SOLAR_ (Equation (12). Like a natural system, solar energy, via the variable Reducer_SOLAR_ (Equation (12), only impacts the top soil layer, called NetSoil_TEMP1_ (Equation (10). NetSoil_TEMP1_ is defined as the following:

(10) $${\text{NetSoil}}_{\text{TEMP}1}={\text{Air}}_{\text{TEMP}}\times {\text{Reducer}}_{\text{SOLAR}}\times {\text{Damping}}_{\text{SOIL}}$$

where daily average air temperature (Air_TEMP_; Table 1), Damping_SOIL_ (Equation (11), and Reducer_SOLAR_ (Equation (12) are multiplied together. AST soil moisture damping was included, yet simplified to only the inversion of each layer’s fraction of volume to volume (*v*/*v*) soil moisture (Layer_SM_; Table 1):

(11) $${\text{Damping}}_{\text{SOIL}}=1-{\text{Layer}}_{\text{SM}}$$

Solar energy was built into the AST-Solar approach by accounting for the proportional relationship between each cell’s solar energy to the landscape cell with the maximum solar energy. At each simulation timestep, the spatially distributed solar energy and the watershed’s maximum solar energy at any location are both used to calculate the solar energy reduction called Reducer_SOLAR_ (Equation (12). Reducer_SOLAR_ represents the localized reduction in soil temperature due to shadowing in relation to the watershed’s maximum ground-level solar energy. The reduction of solar energy is calculated as follows:

(12) $${\text{Reducer}}_{\text{SOLAR}}=1-\alpha \times (1-({\text{Cell}}_{\text{SOLAR}}/{\text{Max}}_{\text{SOLAR}}))$$

where Cell_SOLAR_ is each cell of interest within the VELMA framework, Max_SOLAR_ is the landscape’s maximum solar energy value amongst all landscape cells per time step, and α is a calibration factor. The calibration factor α is a fraction [0.0–1.0, where 0.0 is no solar energy and 1.0 is no change to the solar energy] that allows control over the influence of Reducer_SOLAR_. But, to allow a direct and fair comparison of AST to AST-Solar, calibration factor α was not used in these tests.

VELMA utilizes four soil layers, and the thickness of each layer is customizable. VELMA soil temperature is not directly affected by solar energy, but rather through soil depth attenuation. For layers 2, 3 and 4, the soil temperature (NetSoil_TEMPX_) is calculated using the 2-day running average temperature of the soil layer directly above, plus a reduction by Damping_SOIL_ (Equation (11):

(13) $${\text{NetSoil}}_{\mathrm{TEMPX}}={\text{Soil}}_{\text{AVE\_TEMP}}\times {\text{Damping}}_{\text{SOIL}}$$

(14) $${\text{Soil}}_{\text{AVE\_TEMP}}=({\text{SoilTemp}}_{\left(\text{JDay}\right)}+{\text{SoilTemp}}_{\left(\text{JDay}-1\right)})/2$$

where SoilTemp_(JDay)_ (Table 1) is the current time steps soil temperature, and SoilTemp_(JDay−1)_ (Table 1) is the prior time steps soil temperature. The soil moisture is applied as the Damping_SOIL_ coefficient (Equation (11).

### 2.5. Subroutine Testing

We utilized data from EPA’s O’CCMoN sites to test any change in accuracy and seasonal performance between the AST versus AST-Solar soil temperature subroutines. The O’CCMoN transect dataset provided observed driver data of air temperature, photosynthetic active radiation (PAR) as micromoles/meter^2^/second (μmoles/m^2^/s), and soil moisture as volume to volume at two soil layer depths [23]. EPA’s observed O’CCMoN data helped to compare the AST versus AST-Solar models. Each O’CCMoN site also provided observed soil temperature at two depths. The soil temperature data were used to generate goodness-of-fit metrics against the simulated model results.

#### 2.5.1. O’CCMoN Testing Sites

**Figure 4.**
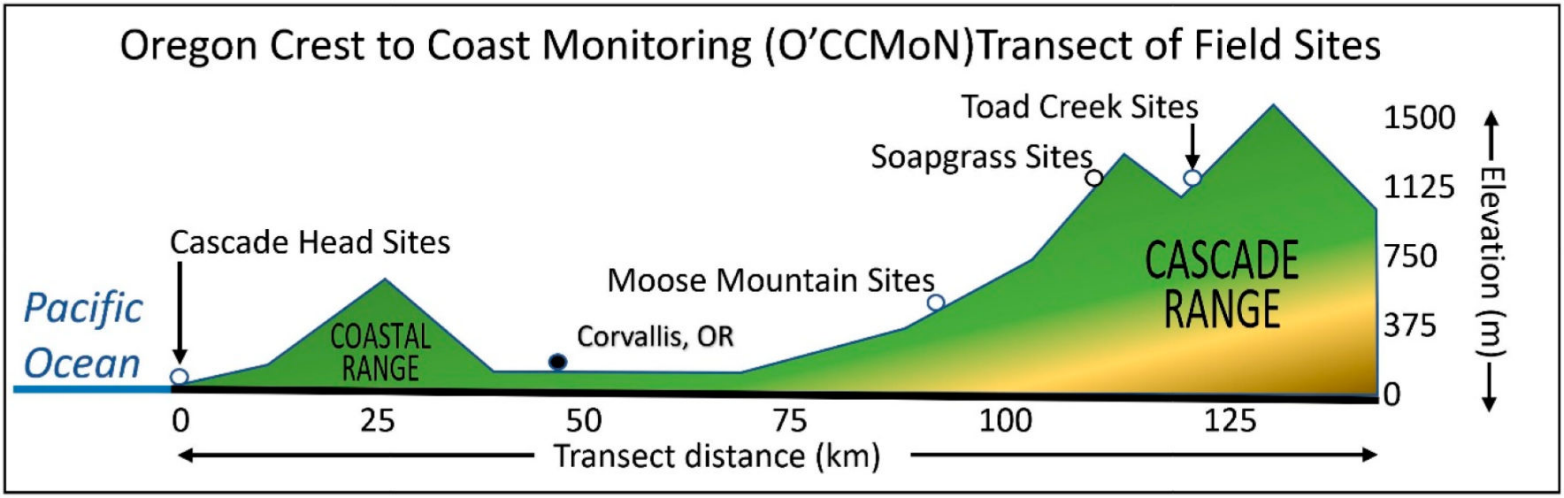
EPA Oregon Crest-to-Coast Environmental Monitoring (O’CCMoN) transit sites with environmental West to East trends and details for the sites used in this study. Figure adapted from the Crest to Coast Overview document [28].

For model testing, the four following O’CCMoN locations were chosen: Cascade Head, Moose Mountain, Soapgrass, and Toad Creek. Each O’CCMoN location contains one forested site and one open clear-cut site (Figure 4).

Overall, these sites span a wide range of elevations and habitat diversities between the coast and the Cascade Mountain snow zone (Table 2).

The Cascade Head open site was installed outside the Cascade Head Experimental Forest and Scenic Research Area-Forestry Sciences Laboratory (EFSRA-FSL) in the managed landscape at an elevation of 157 m (Table 2). The Cascade Head forest site is located 190 m to the northeast in a predominantly Douglas-fir forest at an elevation of 190 m (Table 2). The Moose Mountain, Soapgrass, and Toad Creek sites are positioned on the western side of the Cascades Mountain Range at increasing elevations and experience moderate to extreme weather. The Moose Mountain open site was installed within a forest clear-cut at an elevation of 668 m with the forest site located 460 m to the northeast in a predominantly Douglas-fir forest at an elevation of 658 m (Table 2). The Soapgrass open site was installed within a forest clear-cut at an elevation of 1298 m with the forest site located 1190 m to the northeast in a predominantly Douglas-fir forest also at an elevation of 1190 m (Table 2). The Toad Creek open site was installed within a forest clear-cut at an elevation of 1202 m with the forest site located 471 m to the east in a predominantly Douglas-fir forest at an elevation of 1198 m (Table 2).

The soil temperature probes were all installed in the same manner at all EPA O’CCMoN locations for both the open site and forested site. In each location, two soil temperature sensors were installed at a depth of 15 cm and 30 mm, respectively, from the mineral soil surface, i.e., just below the O-horizon [23]. The testing of the AST and AST-Solar subroutines did not involve any data collection, but rather leveraged the data collected through the EPA O’CCMoN project. These data and the details of field work can be found in the documents at the data repository [23].

#### 2.5.2. AST versus AST-Solar Subroutine Setup

The AST and AST-Solar subroutines were both ran from 1 January 2005 through 31 December 2005 at a daily time step. For each site, the same O’CCMoN observed air temperature and soil moisture data were used as the data drivers for both subroutines [23]. The O’CCMoN data are measured in 30-min intervals, yet the VELMA model functions at a daily time step. To match the VELMA temporal grain, all observed O’CCMoN data were averaged to a 24-h period.

The VELMA spatial framework, per cell, contains four voxel layers; therefore, the AST and AST-Solar subroutines function under this spatial framework. Yet, the O’CCMoN dataset contains soil temperature probe data at only two depths. The AST and AST-Solar soil moisture probe depth variables for layer 1 and 2 were set to match the sensor depths of 15 cm and 30 cm [23]. Since the O’CCMoN data sites contained only two soil moisture probe depths for the sites selected, the AST and AST-Solar voxel layer three and four soil temperature results could not be evaluated and were excluded.

The AST-Solar model utilized the additional solar energy driver data. For the AST-Solar open site simulations, the Cell_SOLAR_ and Max_SOLAR_ variables utilized open site solar energy data. For the AST-Solar forest site simulations, the Cell_SOLAR_ variable utilized the forest site solar data, while the Max_SOLAR_ variable was calculated using the open site solar energy data.

The variable Reducer_SOLAR_ is utilized as a fractional variable scaled from zero to one (Equation (12). This setup allows any solar energy units to be implemented through this method. Cell_SOLAR_ being the solar energy per location and Max_SOLAR_ representing the location with the most solar energy means for an open site, the Cell_SOLAR_ and Max_SOLAR_ values will similar if not the same. In this scenario, Reducer_SOLAR_ will cause minimal to no reduction to the soil temperature. Conversely, forest site Cell_SOLAR_ and Max_SOLAR_ values will be quite different. In this scenario, Reducer_SOLAR_ will cause a reduction in the soil temperature.

## 3. Results

Model results for each of the O’CCMoN sites are summarized in Table 3. Overall, the inclusion of spatially distributed solar energy improved the simulated solar temperature results. Both open and forested sites exhibit gains in accuracy, though the inclusion of spatially distributed solar energy was most beneficial for the forest sites. Below, all data are distinguished using the following attributes: site location, open versus forest environment, and soil layer. O’CCMoN sites with open versus forest locations are listed with abbreviations under “Sites” in Table 3. Soil Layer 1 and Soil Layer 2 are referred to as SL1 and SL2, respectively.

**Figure 5.**
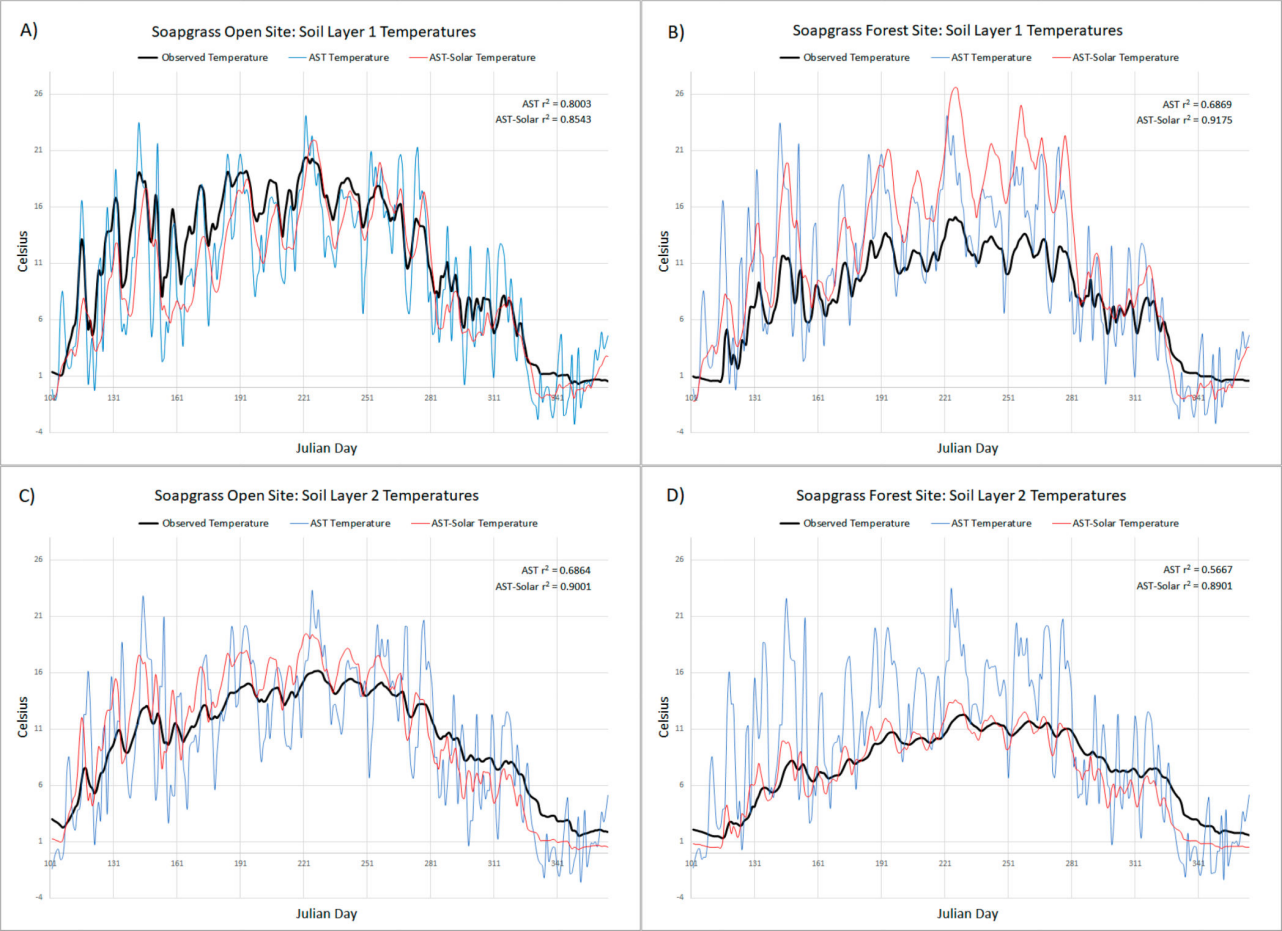
All panels compare observed data for the year 2005, AST model results, and AST-Solar model results. (Panel **A**) Soapgrass Open Site Soil Layer 1 temperature results. (Panel **B**) Soapgrass Forest Site Soil Layer 1 temperature results. (Panel **C**) Soapgrass Open Site Soil Layer 2 temperature results. (Panel **D**) Soapgrass Forest Site Soil Layer 2 temperature results. Simulation r^2^ values are calculated using Julian days 101–365. Ignoring the first 100 days prevented the AST model from being penalized for its 100-day phase lag, which only occurs during the first year of simulation.

The performance of the AST-Solar model at the Soapgrass site increased for soil layers 1 and 2 at both the open and forest sites compared to the AST model, but especially for soil layer 2 (Table 3). Specifically, the SGO-SL1 performance increased from a r^2^ of 0.80 to 0.85 (Table 3; Figure 5A), while the SGO-SL2 performance increased from a r^2^ of 0.69 to 0.90 (Table 3; Figure 5C). The SGF-SL1 performance increased from a r^2^ of 0.69 to 0.92 (Table 3; Figure 5B), while SGF-SL2 performance increased from a r^2^ of 0.57 to 0.89 (Table 3; Figure 5D).

The results among all sites are unique for each site, though the seasonal pattern and increased performance are similar over the year. Since the patterns are similar for each of the different sites, only the Soapgrass site results are graphically represented. Due to the 100-day Phase_LAG_ (spin-up) requirement of the AST model, only Julian days 101 through 365 are graphically represented.

The performance at Cascade Head (CH) increased for the AST-Solar subroutine compared with the AST subroutine for soil layers 1 and 2 for the forest site, but the performance only improved soil layer 2 of the open site. The open site soil layer 1 was the only simulation that exhibited a decrease in simulated versus observed agreement by decreasing from a r^2^ of 0.83 to 0.77. In contrast, the CHO-SL2 performance increased from a r^2^ of 0.71 to 0.95. All CH14 simulations improved. The CH14-SL1 performance increased from a r^2^ of 0.74 to 0.87, while the CH14-SL2 performance increased from a r^2^ of 0.71 to 0.94.

The performance of the AST-Solar subroutine compared with the AST subroutine at the Moose Mountain (MMO) increased for soil layers 1 and 2 at both the open and forest sites, particularly for soil layer 2. The MMO-SL1 performance increased from a r^2^ of 0.81 to 0.92 (Table 3). The MMO-SL2 performance increased from a r^2^ of 0.67 to 0.93 (Table 3). The MMF-SL1 performance increased from a r^2^ of 0.89 to 0.93 (Table 3). The MMF-SL2 performance increased from a r^2^ of 0.70 to 0.94 (Table 3).

The performance of the AST-Solar subroutine over the AST subroutine at the Toad Creek increased for soil layers 1 and 2 at both the open and forest sites. The TCO-SL1 performance increased from a r^2^ of 0.82 to 0.83, while the TCO-SL2 performance increased from a r^2^ of 0.73 to 0.92 (Table 3). The TCF-SL1 performance increased from a r^2^ of 0.83 to 0.90, while the SGF-SL2 performance increased from a r^2^ of 0.64 to 0.89.

## 4. Discussion

VELMA’s original soil temperature model functioned well without the inclusion of spatially distributed solar energy (see Table 3; Figure 5), yet the inclusion of spatially distributed solar energy can provide significant improvements to simulated ecological processes driven by solar energy. The inclusion of local ground-level solar energy data improved VELMA’s AST subroutine simulations of soil temperature from more than one perspective. First, the observed versus modeled comparisons for all sites improved, with one exception. The only exception was the CHO site, which was a landscape anomaly amongst the sites due to the station existing within a regularly maintained grass lawn at the headquarters of the research area (i.e., Cascade Head EFSRA-FSL). The TCO site received the smallest soil temperature modeling improvement with SL1 r^2^ increasing from 0.82 to 0.83, yet the TCF site showed a significant SL2 improvement with a r^2^ increase of 0.73 to 0.92. The largest single layer improvement was observed at the SGF site with the SL1 r^2^ increasing from 0.69 to 0.92 and SGF-SL2 r^2^ increasing from 0.57 to 0.89. It is worth reiterating that though the AST-Solar subroutine has a calibration parameter, no calibration was applied when simulating any of the sites to ensure the AST to AST-Solar estimates of soil temperature were a fair comparison of the subroutine performance.

Beyond the r^2^ goodness-of-fit metrics, the daily variability in the modeled AST-Solar soil temperature data was reduced. This can be seen in all the graphs presented above by comparing how the AST subroutine demonstrated a repeated overestimation and underestimation of soil temperature compared to the observed data. However, the AST-Solar subroutine’s noise was greatly reduced. This noise pattern in AST was due to the significant influence from the daily average air temperature driver data. Therefore, the static equations that provided oscillatory proxies for solar energy did not fully parallel the environmental phenomena of solar energy. In part, this disparity explains the resulting noisy estimations of soil temperature when solar energy was not directly included in the subroutine.

VELMA’s AST soil temperature subroutine required a 100-day soil temperature simulation spin-up period. This time lag allowed for a sufficient temporal delay in the driver data utilized by Equation (6) (note that this time lag is only required for the first year of multi-year AST simulations). The purpose of the lag time was to account for the seasonal weather influence on the soil column. For the new AST-solar subroutine, this lag was no longer required due to the addition of localized daily solar energy data interacting with the existing soil moisture within layer 1.

Both the AST and AST-Solar subroutines do not model the insulation effects of snow pack [4]. Further work could include snow depth and its insulative effects on soil temperature. This would improve the soil temperature estimates in the winter, due the insulation of heat from the snow pack preventing the soil temperature from getting colder or even freezing. For the AST-Solar model, this may further improve performance with observed data (Figure 5, panels A–D) for days where snow pack persisted at Soapgrass (Figure 2) as well as goodness-of-fit metrics for the other O’CCMoN monitored sites. Similarly, further improvements in AST-Solar soil temperature estimates may be possible by including VELMA predictions of surface detritus (dead leaves and wood) and ground-level leaf biomass (proxy for leaf area index) that contribute to near-surface shading of mineral soil surfaces in open and forest sites.

The subroutine performance improvements reported here are due to the influence of local solar energy, which alters the resulting soil temperature. The prior soil temperature model predominantly utilized average air temperature as a proxy for energy, which is commonly done in watershed models. Though VELMA is a spatially distributed model, the default weather model is driven by single site location climate data. This setup resulted in homogeneous average air temperature across the simulated watershed. This is true for all forest cells and bare open prairie or forest clear-cut cells alike. By including local solar energy representation, the subsequent modeling of soil temperature was enhanced due to the improved model representation of the real world. This mainly was accomplished through the inclusion of the environmental variable solar energy that causes direct and significant influence on the phenomena soil temperature.

## 5. Conclusions

Watershed models are widely used to simulate the effects of land use change on the environment and the quantity and quality of hydrologic components throughout a watershed. In this paper, we demonstrated that local solar energy information improved soil temperature modeling estimates simulated by a soil temperature subroutine within a larger ecohydrological watershed model. These models were compared with observed data for soil temperatures at two depths within both open and forested environments among four observed data sites (i.e., EPA’s O’CCMoN transect data) [23].

Overall, by including explicit information regarding the spatial distribution of solar energy across a landscape, watershed models can better capture the spatiotemporal variations of soil temperature in both forested and open sites. Therefore, researchers that utilize spatially distributed or semi-distributed mechanistic watershed models should consider incorporating spatially explicit solar energy models (e.g., Penumbra [30,31]) or other spatially heterogeneous descriptions of ground-level solar energy to better represent energy exchange at the surface. This is especially true when modeling discrete landcover types such as forested, open, water, and agricultural cover and when modeling the impacts of riparian shading on soil temperature and stream temperature, as well as the effect of solar energy on fish habitat.

Finally, while we presented improvements of a soil temperature subroutine within an ecohydrological model, other subroutines can also benefit by the inclusion of spatiotemporal representations of ground-level solar energy. The integration of local solar energy information with watershed models and all their subroutines could potentially benefit several processes, such as snow melt, water temperature, and plant growth via photosynthesis. Integration of spatially explicit ground-level solar energy models with environmental models can provide dynamic feedbacks between other environmental processes, such as tree growth and shade. As tree growth is simulated within watershed models, their heights could be transferred back to the ground-level solar energy model in a dynamic mechanism, which then would alter the amount of solar energy that is intercepted by the canopy and does not reach the ground. This dynamic integrated modeling approach could be extremely beneficial for looking at the long-term effects of planting riparian buffers and determining the duration required for stream temperatures to be reduced by some threshold. Because solar energy is amongst the most impactful environmental variables in natural and managed ecosystems, further investigations would greatly benefit from the application of watershed-scale models that are dynamically coupled with spatially-explicit solar energy models.

## Figures and Tables

**Table 1.** AST and AST-Solar subroutine input variables.

**AST Variables**	**Descriptions**

Air_AVETEMP_	Fixed value of 8.2 (°C)
Air_LAG_	Historic air temperature derived from the Phase_LAG_.
LS_DEPTH_	Soil column depth to center (mm) per layer of interest
LTD_ACCUMULATION_	Summation of the thermal deltas per layer of interest
Soil_BELOW_	Soil layer below the current layer being calculated

**AST-Solar Variables**	**Descriptions**

Air_TEMP_	Daily average air temperature in the open site
Soil_AVE_TEMP_	Two-day running average (°C)
Reducer_SOLAR_	Derived value from the input solar energy data
Layer_SM_	Volume to volume soil moisture level
SoilTemp_(JDAY)_	Current time steps soil temperature value
SoilTemp_(JDAY−1)_	Prior time steps soil temperature value

**Table 2.** O’CCMoN Open and Forest Site Characteristics.

Site Name	Elevation (m)	Vegetative State	Annual Rainfall (cm)	Tree Height (m)	Soil Parent Material

Cascade Head: Open (CHO)	157	Lawn		-	
			
Cascade Head: Forest (CH14)	190	Alder Douglas-fir Sitka Spruce	200–250	50–60	Marine Sediment

Moose Mountain: Open (MMO)	668	Clear-cut	150–180	-	Volcanic
	
Moose Mountain: Forest (MMF)	658	Douglas-fir		50–60	

Soapgrass: Open (SGO)	1298	Clear-cut	180–200	-	Volcanic
	
Soapgrass: Forest (SGF)	1190	Douglas-fir		60–70	

Toad Creek: Open (TCO)	1202	Clear-cut	180–200	-	Volcanic
	
Toad Creek: Forest (TCF)	1198	Douglas-fir		50-60	

Note: All information in this table was obtained from the O’CCMoN dataset documentation, except the annual rainfall which was obtained through the PRISM 1981–2010 annual rainfall normals [29].

**Table 3.** VELMA-AST and VELMA-AST3 O’CCMoN results.

O’CCMoN Location	Sites	Soil Layer 1		Soil Layer 2	

		AST (r^2^)	AST3 (r^2^)	AST (r^2^)	AST3 (r^2^)

Cascade Head	Open Site (CHO)	0.83	0.76	0.71	0.95
	Forest Site (CH14)	0.74	0.87	0.71	0.94

Moose Mountain	Open Site (MMO)	0.81	0.92	0.67	0.93
	Forest Site (MMF)	0.89	0.93	0.70	0.94

Soapgrass	Open Site (SGO)	0.80	0.85	0.69	0.90
	Forest Site (SGF)	0.69	0.92	0.57	0.89

Toad Creek	Open Site (TCO)	0.82	0.83	0.72	0.92
	Forest Site (TCF)	0.83	0.90	0.64	0.89
